# Design of an Integrated Clinical Research Informatics System for a Multi-Centre and Multi-Visit Prospective Birth Cohort Study

**DOI:** 10.3233/SHTI220045

**Published:** 2022-06-06

**Authors:** Ratchaneewan Sinitkul, Richard J Maude, Ussanai Nithirochananont

**Affiliations:** a Department of Paediatrics, Faculty of Medicine Ramathibodi Hospital, Mahidol University, Bangkok, Thailand; b Mahidol-Oxford Tropical Medicine Research Unit, Faculty of Tropical Medicine, Mahidol University, Bangkok, Thailand; c Centre for Tropical Medicine and Global Health, Nuffield Department of Medicine, University of Oxford, Oxford, United Kingdom; d Toxicology Department, Centre for Radiation, Chemical and Environmental Hazards, Public Health England, Chilton, United Kingdom; e Harvard T.H. Chan School of Public Health, Harvard University, Boston, MA, USA; f Geo-Informatics and Space Technology Development Agency, Chonburi, Thailand

**Keywords:** Clinical Research Informatics, Medical Informatics, Health Information Management

## Abstract

To conduct a multi-centre prospective study over more than one year requires an efficient system that can synchronise collection of data from several sources in real-time and facilitate remote data management. This paper describes the design and use of an in-house data collection and sample information management system that was used in a prospective birth cohort study in Thailand. Participants were enrolled from three hospitals and were required to visit their respective hospital and complete self-administered questionnaires (SAQ) at every visit. The in-house informatics system required integration of the data collection streams that can handle three different types of data (SAQ, clinical record, and laboratory sample tracking). The system has been implemented in the pilot phase of a birth cohort study and has demonstrated its usability for further application to an expanded study.

## Introduction

One aspect of clinical research informatics (CRI) concerns the development of information systems to support the optimal design and conduct of clinical research studies [1] including, for example, improving information capture and enhancing data integration. In this manner, a data collection and management system can be regarded as a part of a CRI system.

Collecting data in a multi-centre and multi-visit birth cohort research study, particularly in the environmental health field, is challenging due to the vast amount of information to be captured from multiple sources over a long period of time, e.g. participants, specimens and healthcare records. It can be a substantial burden for research staff taking up a lot of their time to enter data and monitor progress, data quality and completeness. Therefore, a data collection system should be designed to minimise these difficulties. Developing an electronic or web-based system that combines all the required data collection systems into a single comprehensive system could address the above issues.

In the literature, researchers have developed electronic data collection and management (EDCM) systems using a variety of approaches, e.g. a web-based system or a standalone system, to support their study. Many have relied on REDCap [2-4], Kobo Toolbox or Open Data Kit (ODK) whilst others have developed their own in-house system that could provide more flexibility to meet the particular needs of their study, [5-7]. Some groups have used a hybrid system that combines REDcap or ODK with other software, either commercial or in-house [8; 9]. There are also commercial solutions that are generally costly to implement. We were unable to find a suitable low cost solution for the multiple sources of data collected in a complex study such as our environmental health birth cohort study so we chose to develop our own.

Research Electronic Data Capture (REDcap) [10] is a widely used and well-known web application for building and managing online surveys and databases. However, its one-to-one relationships model is less flexible to implement one-to-many (parent-child) relationships as such required by some data, e.g. tracking specimen and its aliquots. In addition, this study requires a data capture system with multiple users with different levels of access to minimise risk of compromise of data security. It was also desired to have a system that can be used to monitor progress of each component of the study and the project as a whole. Therefore, an in-house web application for data collection and management was developed. It provided a comprehensive EDCM system. This integrated system can collect and manage data populated from self-administered questionnaires (SAQ), clinical records, and biospecimen collection and storage. These three data sources are essential for a birth cohort study especially in the environmental health field and are entered in to the system by different actors including participants (research subjects), investigators (physicians), and laboratory technicians. The system was also used for project monitoring, and dynamically generating basic updated statistics as the study progressed. The EDCM was used at multi-centres in Thailand (Ramathibodi Hospital - the Medical school and quaternary hospital in Bangkok, Rayong Hospital and Samut Prakarn Hospital; both provincial, tertiary health care hospitals in industrialised area).

In this paper, we describe a design approach for developing this bespoke clinical research informatics system. We demonstrate the application of this approach to support the pilot phase of a birth cohort study in Thailand with emphasis on data collection, study monitoring, data security and lessons learnt.

## Methods

### Study Protocol

**Figure 1:**
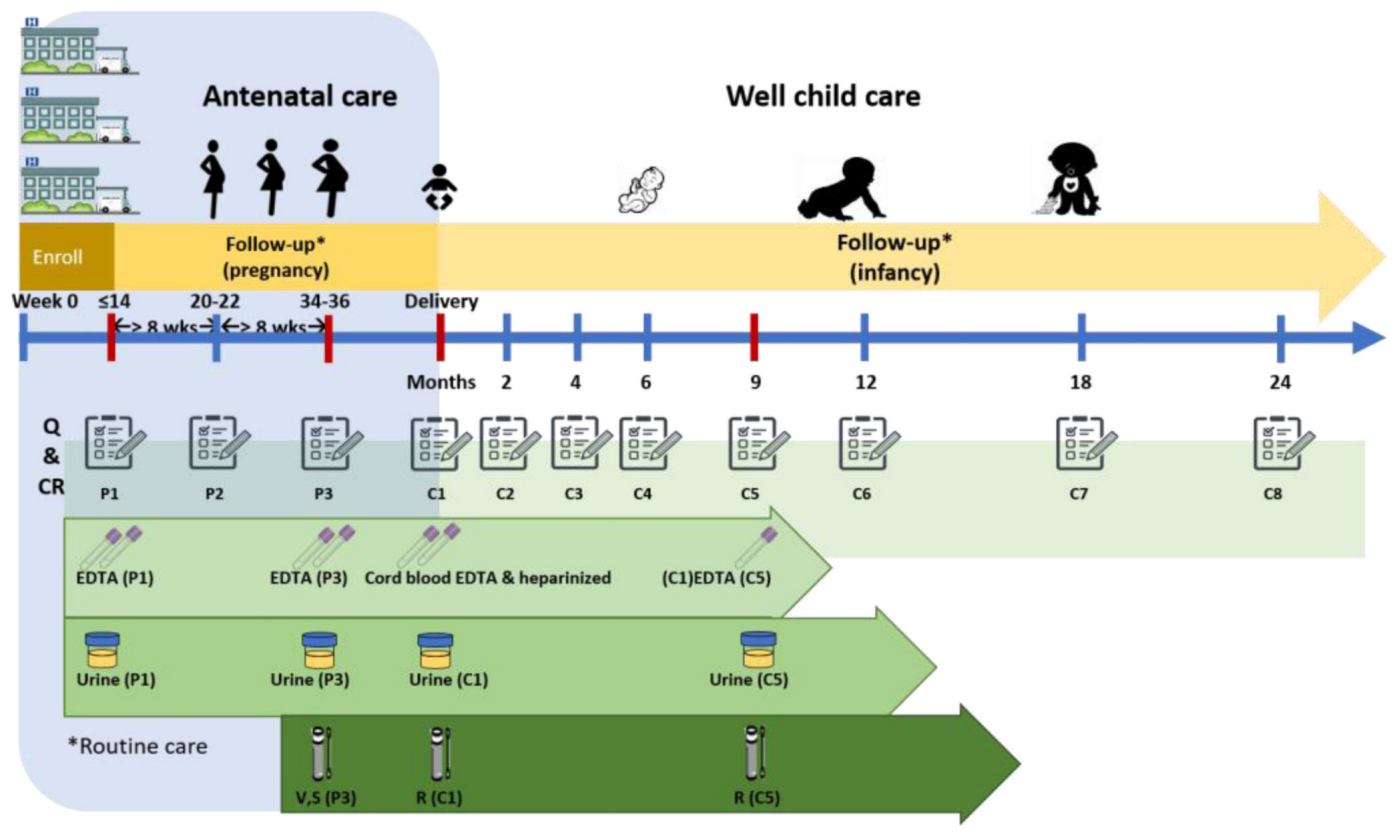
Summary of study protocol for the pilot birth cohort study on children’s environmental health in Thailand

The protocol for the study was granted ethical approval by the Institutional Review Board, Faculty of Medicine Ramathibodi Hospital in 2018. The study protocol is summarised in Figure 1. In brief, a total of 200 pregnant women were enrolled from three hospitals in Thailand at Ramathibodi hospital, Samut Prakarn hospital, and Rayong hospital. The participants were followed up during the pregnancy period until their expected child/children reached two years old. The data collection included repeated SAQ every 2-3 months, clinical record data, and biospecimen collection and storage. The study was conducted from April 2018 onwards and is expected to finish in December 2021.

### Design Procedure

Design procedure in this paper consists of three steps: 1) gathering requirements to select technology and development tools 2) analysing workflow in the study to define components in the system, and 3) designing system components.

### Requirements

The design of the system began with gathering requirements from the research staff. In the iterative meeting with the development team, three key requirements based on the study protocol were identified. The first requirement was multiple sites and multiple participant visits. the study sites were separate hospitals in distant major industrial cities and participants required to have eleven visits during their participation. Data collected by these hospitals at all visits was needed to be shared in real-time whilst avoiding duplicating copies of the data. Second, the system should be intuitive and user-friendly so that it takes minimum time for any actor, especially participants, to learn to use. Lastly, participants are expected to participate in the study for at least 2.5 years from their first trimester until their child/children is/are two years old. The system should also be able to track and monitor progress, both of individual participants as well as the overall project.

### Workflow

As shown in Figure 1, the study required all participants to have eleven visits at the same hospital. The first three of these visits (P1, P2 and P3) occurred when participants were pregnant. The rest of the visits (C1, C2, C3, C4, C5, C6, C7, and C8) were after delivery and participants’ children being at newborn, 2-, 4-, 6-, 9-, 12-, 18-, and 24-months of age. All these eleven visits corresponded to their regular appointments with the hospital. During these visits, data related to a participant were categorised into three groups based on means of collection: 1) questionnaire 2) clinical examination and investigation and 3) biospecimens.

The SAQ was intended for participants to complete themselves during each of the 11 visits over 30 months of the prospective study. The first 3 visits were for pregnant participants whilst the remainder were for their children. For pregnant women, each visit consisted of five sections of questions, 1) personal information, 2) health background, 3) environmental exposure, 4) occupational risk, and 5) food frequency questionnaires (FFQ). The children questionnaires focussed on raising children with each visit having four sections, 1) raising information, 2) developmental surveillance, 3) developmental promotion, and 4) additional raising problems (opened-questions).

The Clinical Record Form (CRF) was used to record participant information in six sections: 1) history, 2) physical examination, 3) investigation, 4) prenatal ultrasound, 5) diagnosis, and 6) medication. The examination for children differed between visits. For example, in the first visit for newborn children, they were examined for both delivery and post-delivery conditions. In addition, they were also further examined for allergies. The examination was performed by the of the participants’ physician. The study collected this information from the medical record and entered it into the CRF.

In the biospecimen information, lab technicians recorded details of specimens collected from participants and their children. Specimens were classified into two stages of processing: pre-storage and storage specimens. The pre-storage were the original specimens taken from participants (7 types) which were then transferred into an appropriate container for freezing or further processing then freezing (10 types).

### System Components

**Figure 2:**
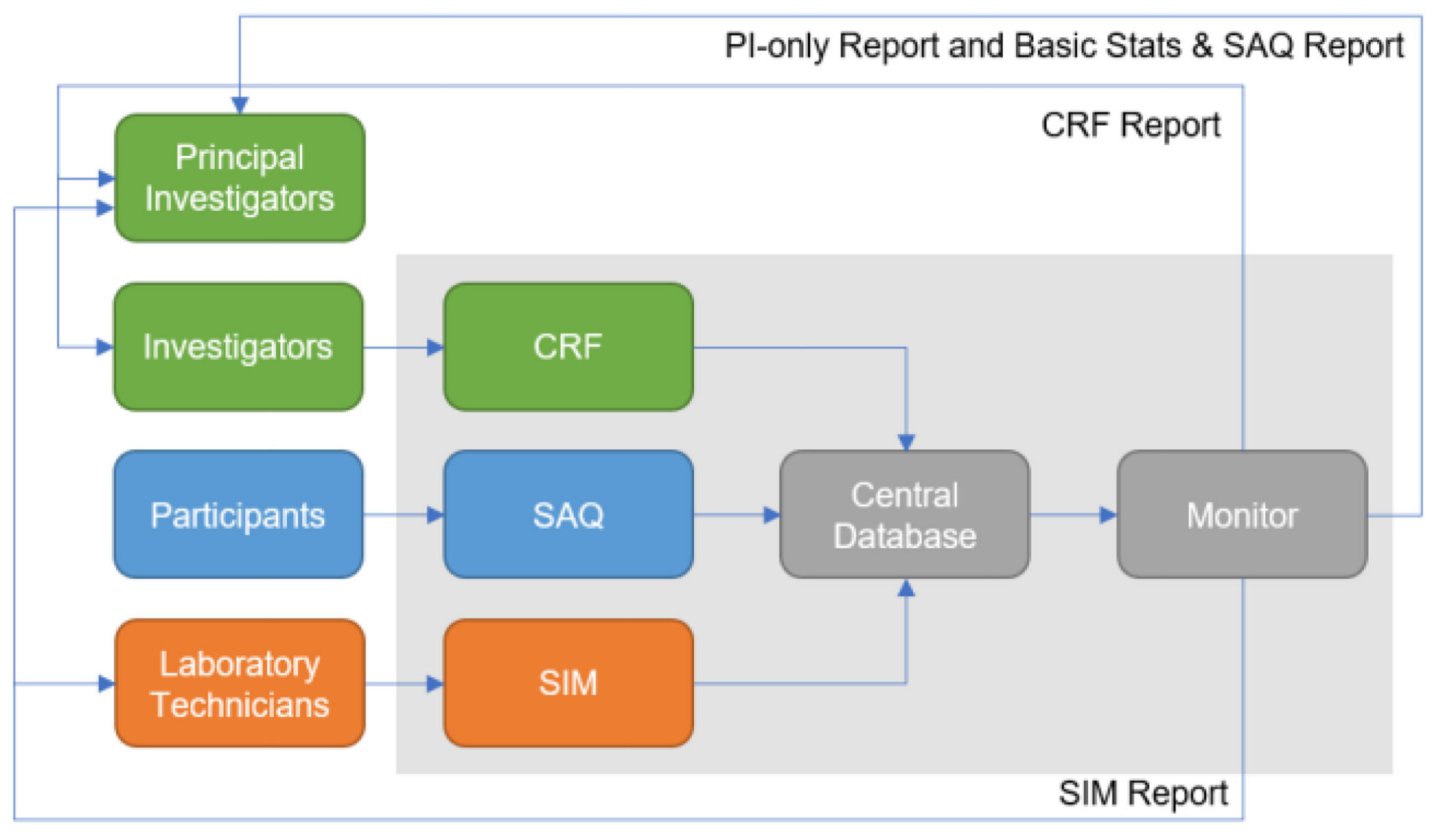
Designed system and its components

In brief, based on the workflow, the designed components (Figure 2, light grey box) were the SAQ, the examination recorded in the CRF, and specimen information during its journey from participant until storage handled by Sample Information Management (SIM). The system also required a central database to stored the data as well as a component to provide tracking and monitoring of study activities.

## Results

### Development Tools

Based on the requirements gathered in the design meeting, it was decided to develop the system as a web application with domain name *wicare.info.* A web application is a client-server computer program that runs on a web browser. For ease of use and convenience, the developed system was intended for use on both desktop computers and mobile phones, and a variety of web browsers. Therefore, the web application was developed using Hypertext Markup Language version 5 (HTML5) so that it could be supported by major web browsers including Chrome, Internet Explorer, Safari, and Firefox. Also, PHP and JavaScript, which are scripting languages, were used to enable dynamic and interactive features in the web application, e.g. live validation of data entered. MariaDB, a relational database based on MySQL, was used to store data collected by the application. The web application manages (e.g. add, delete, or update) this database using Structured Query Language (SQL) commands which were provided by PHP.

### Roles in Accessing System

Participants, investigators, and laboratory technicians were granted access with different permissions and could only access the part of the system to which they had been assigned to enter data. The authentication system verified user’s permissions and took them to the appropriate part of the system after login. For reviewing raw data, investigators and laboratory technicians are restricted to the data collected from participants within their hospital. Viewing of data from other hospitals is not permitted. Only the principal investigator can access the entire system and view reports or retrieve data from multiple hospitals or multiple systems without any restrictions.

### Data Collection and Management

All data capture in the pilot was done using online forms. However, the workflow differed between the three components (SAQ, CRF and SIM) due to different roles of the users who entered the data.

As required by the data collection workflows, the system consists of three collection tools for each individual data type. A database was also required to store these data, which can be either a central database with individual tables or separate databases. Due to different metadata of the required data, a database component was designed based on a relational model in which each data type can be linked to the others using a unique participant ID and visit code, e.g. R00001 and P1, respectively). The data collection was also conducted longitudinally; therefore, a monitoring component was required to track activities.

Questionnaires and clinical data were captured once per visit whilst specimens could be captured more than once in a single visit as multiple types of specimen were acquired. Each specimen also had its own metadata, e.g. type of specimen, date acquired, and participant ID. Therefore, all the data had to be stored in separate tables. These tables were designed to contain structured data formats as all data fields to be collected must have assigned values covering all conditions including missing or skipped fields. As a result, relational databases (MySQL) are suitable for storing these data.

All data have to be stored in the central database so that they can be retrieved to generate reports and exported for further analysis. As shown in Figure 2, the system consists of five components: SAQ, CRF, SIM, Monitor, and Central Database. The first three parts were used to collect their corresponding data and the monitor was used to retrieve the data and generate up-to-date reports. All data were stored in the individual tables in the central database.

### Tracking and Monitoring Progress

The system allows investigators and laboratory technicians to review the data they have entered through the monitoring component. For the principal investigator, in addition to the raw data, the monitor also contains data summaries and descriptive reports. For example, the report used for monitoring individual participants includes status of completing questionnaires and estimated date of delivery (EDD). In children, appointment details (e.g. date, and place) for the next visit can also be shown. Those reports are automatically generated and updated in near real-time as soon as the new data are added to the database. Data shown in the monitor can be exported in comma-separated values (CSV) format for further analysis in statistical software.

### Data Security

**Figure 3:**
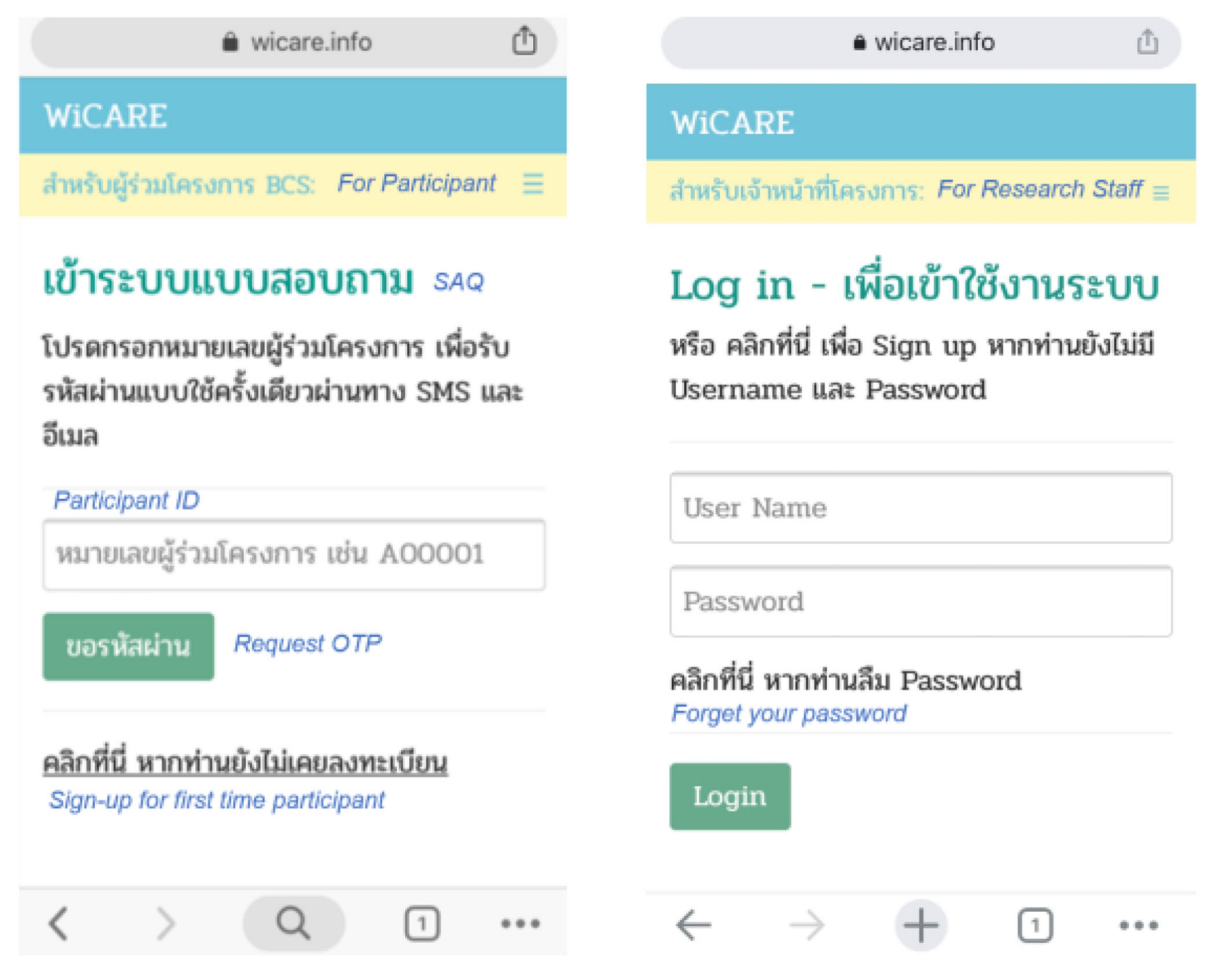
Log-in screenshots on a mobile device (left) for participants and (right) for research staff. The italic text is for illustrative purposes and is not shown in the actual system.

The system is protected by a password, as illustrated in Figure 3. For participants, they use a one-time password (OTP) sent by the system via SMS and email. The OTP is set to expire if not used within one hour. The system automatically logs out if there is no response from participants within 60 minutes to prevent access by unauthorised users. For investigators and laboratory technicians, they use their own pre-defined password that is provided during registration. After successfully logging in, participants, investigators, and laboratory technicians are brought to different landing system pages. Only the principal investigator can access all areas.

To protect the system (a web application) from cross-site scripting, which is injecting harmful code into the original code, all input fields use sanitisation methods.

The web server, which stores the entire web application, is a cloud server that provides stability and security. It is also protected by a firewall and anti-virus software, and is backed up daily. The communication link between the web server and participants’ web browser is encrypted using Secure Sockets Layer (SSL). This SSL ensures that all data passing through these links remains private.

### System Functional Verification

The application was verified for its functionality to ensure that it can work properly to store and retrieve data. The functional verification was done by two testers: the system developer, and the principal investigator. Each tester used one of two different pre-defined datasets to enter to the application. Data stored in the database were retrieved and compared to the original datasets. The verification was iterated with new pre-defined datasets until data was stored and retrieved correctly.

### Lessons Learnt

#### Questionnaire Output Data

In our first iteration of the SAQ development, due to some unclear communication about the requirements between research staff and the development team, the SAQ output was stored in the database as text which cannot be analysed directly in statistical software. To seamlessly analyse data in statistical software, the questionnaire responses were converted into numeric values before storage in the database. Therefore, we defined a data dictionary to assign numeric value to response options and then added PHP code, based on this data dictionary, to implement a conversion.

#### One Time Password

The SAQ initially verified participants using OTP. However, in the first few months of the study we found that some participants did not receive their OTP and could not login to administer the questionnaires. We investigated the problem and found that some mobile network providers may treat the OTP from the system as a spam advert, and as a result they blocked the OTP.

We have modified the system so that it can also send the OTP to participant’s email addresses which they provided when registering on the system. This was found to resolve the issue for some participants, but some still did not receive the OTP. This was because those participants could not remember the password to access their email. To address this issue. we further modified the system to support participants’ pre-defined passwords. Since then this issue was no longer reported.

#### Validation Constraitns for Questionnaires

Our SAQ implemented extensive form validation to ensure the quality of the data entered. However, some participants reported that this made time-consuming to complete the SAQ. In the expanded study, this issue should be reviewed and the system should be modified to be make it more user-friendly.

#### Next Steps

Having completed the pilot, we are adapting the system for use in a larger birth cohort study.

#### Potential for Adaptation for Other Studies

This system was designed so it can be easily adapted for use in other studies which have similar data and study monitoring needs. User authentiation scripts could be used straighforward. However, efforts required for modifying questions and form labels. We aim to make the code publicly available upon completion.

## Discussion

We followed the proposed design approach to develop an in-house EDCM for data collection and study monitoring. This was been implemented to ably support the pilot phase of a birth cohort study on children’s environmental health in Thailand. Despite the system being thoroughly designed and tested, unforeseen issues still occurred during the implementation including user verification steps and questionnaire validation.

REDcap has been widely use for research data capture and data management and it freely available to consortium members [11]. However, its database provides a flat-structure model (one-to-one relationships) [10], which is less flexible for data collections require one-to-many or parent-child relationships, e.g. sample tracking with specimen- and aliquot-level data. In such case, relational model, as also employed in our system, is more flexible to handle. Our EDCM, which uses open platform program script, is an alternative way for the researchers to efficiently collect and manage research data. In addition, it provides additional functionality for self entry of data by participants compromising data security, capabilities for which are limited in REDcap [12].

In accordance with the increasing trend of the general use of smartphones and the internet connected devices in Thailand [13; 14], we used OTP via participant’s mobile number and participant’s email addresses for registration to the system after opt-in. However, this did not work for some participants due to blocking by the mobile network provider or participants forgetting their email login details. Therefore, we suggest that multiple different approaches should be considered during the system design to meet the needs of different participants and get around these problems for registration, login and completing data collection forms.

The advantage of electronic data capture over paper-based collection includes ease of data validation [11]. However, we found challenges with validation of the complex form used in the SAQ which reduced user-friendliness. Balancing between questionnaire validation and user friendliness should be carefully considered to prioritise which validation checks to include during questionnaire development to minimise this issue.

## Conclusions

This paper presents the design of a clinical research informatics system for supporting a birth cohort study in Thailand. The design procedure comprised gathering requirements, analysing study workflow and designing system components. The end result was a bespoke web application that combines self-administered questionnaires, clinical record forms, sample information management, study progress monitoring and basic analytics. This kind of system could help to efficiently manage the research data in other prospective studies.
